# Eco-friendly synthesis of gold nanoparticles using *Equisetum diffusum* D. Don. with broad-spectrum antibacterial, anticancer, antidiabetic, and antioxidant potentials

**DOI:** 10.1038/s41598-025-02450-9

**Published:** 2025-06-02

**Authors:** Nasir Assad, Marzia Batool Laila, Muhammad Naeem Ul Hassan, Muhammad Fayyaz ur Rehman, Liaqat Ali, Muhammad Mustaqeem, Barkat Ullah, Muhammad Nauman Khan, Majid Iqbal, Sezai Ercişli, Abdullah A. Alarfaj, Mohammad Javed Ansari, Tabarak Malik

**Affiliations:** 1 Institute of Chemistry, University of Sargodha, Sargodha, 40100 Pakistan; 2 Department of Botany, Islamia College Peshawar, Peshawar, 25120 Pakistan; 3 Biology Laboratory, University Public School, University of Peshawar, Peshawar, 25120 Pakistan; 4 Key Laboratory of Ecosystem Network Observation and Modeling, Institute of Geographic Sciences and Natural Resources Research, Chinese Academy of Sciences, 11A, Datun Road, Chaoyang District, Beijing, 100101 China; 5 University of Chinese Academy of Sciences, (UCAS), Beijing, 100049 China; 6 Department of Horticulture, Agricultural Faculty, Ataturk University, Erzurum, Türkiye; 7 HGF Agro, Ata Teknokent, Erzurum, Türkiye; 8 Department of Botany and Microbiology, College of Science, King Saud University, Riyadh, 11451 Saudi Arabia; 9 Department of Botany, Hindu College Moradabad, (Guru Jambheshwar University Moradabad), Uttar Pradesh, 244001 India; 10 Department of Biomedical Sciences, Institute of Health, Jimma University, Jimma, 378 Ethiopia; 11 Division of Research & Development, Lovely Professional University, 144401 Phagwara, India

**Keywords:** Green synthesis, Gold nanoparticles, *Equisetum diffusum*, Anticancer activity, Antidiabetic activity, Antioxidant activity, Biological techniques, Biotechnology, Plant sciences

## Abstract

The present study reports, the eco-friendly synthesis of gold nanoparticles (AuNPs) using *Equisetum diffusum* D. Don. extract, a medicinal plant known for its therapeutic properties. Phytochemicals present in the extract served as reducing and stabilizing agents for synthesizing stable AuNPs with an average size range of 68.8 nm. The biosynthesized AuNPs were characterized using UV–vis spectroscopy, FTIR, XRD, SEM, EDX, and dynamic light scattering (DLS) methods, confirming their stability, morphology, and crystalline nature. The green synthesized ED@AuNPs exhibited promising biological activities, including broad-spectrum antibacterial activity against both Gram-positive and Gram-negative bacteria, with inhibition zones from 24 to 37 mm. The anticancer activity was assessed through an MTT assay against hepatic carcinoma (HePG2) cells, revealing dose-dependent cytotoxicity with maximum inhibition at 200 µg/mL (47.62%). Antidiabetic activity was demonstrated by starch hydrolysis and enzyme kinetics, with significant α-amylase inhibitory activity up to 70.85%, comparable to the standard drug Acarbose. Moreover, antioxidant activity was conformed through FRAP and DPPH assays, indicating strong free radical scavenging activity and reducing ability. The study demonstrates the potential of biosynthesized ED@AuNPs as multifunctional agents with applications in biomedicine, particularly in antibacterial, anticancer, antidiabetic, and antioxidant therapies, offering an eco-friendly and sustainable approach for nanoparticle synthesis.

## Introduction

The field of nanotechnology has emerged as an important protagonist in multiple domains, including biomedical research^1–3^, catalytic processes^4^, metal sensing^5^. Nanoparticles (NPs) have drawn a lot of attention due to their large surface-to-volume ratio and extremely small size, which falls in the nanometer range^6,7^. Nanotechnology has the potential to make significant contributions to the domains of drugs, agriculture, and curative diagnostics^8^. Among metal NPs, gold nanoparticles (AuNPs) are the subject of greater research, because of their distinctive medical, mechanical, electrical, size-dependent, and magnetic properties^9^. Antioxidant, antibacterial, targeted drug delivery, anticancer, antimycotic, and other uses have been made of metals such as silver, gold, and selenium and their oxides. AuNPs are synthesized by physical, chemical, and biological synthesis techniques^10^. As a result of the physical and chemical methods need specialized equipment and synthesis settings, use of hazardous chemicals, high energy consumption, and the release of toxic chemicals with hazardous byproducts, considered as unfavorable^3,11^. In contrast, the green synthesis methods are thought to be non-toxic, inexpensive, and ecologically benign^12,13^.

Due to their cost-effective, lack of toxicity, and green chemistry foundation, the biological synthesis of AuNPs employing plants, algae, fungi, and microbes, minimizes the drawbacks of the aforementioned approaches^14^. The biosynthesis of AuNPs using plants is attracting more researchers since the phytoconstituents of plant extract act as both reducing and stabilizing agents. Many plant extracts have potent phytoconstituents that facilitate sustained AuNPs synthesis for drug delivery, antibacterial, antioxidant, anticancer, and photochemical dye degradation. As reported in earlier research, multiple plant materials have been utilized to synthesized NPs^15^. It was observed that stable AuNPs synthesized using *B. hispida* peel extract with antibacterial and anticancer properties^16^. Additionally, it has been reported that curcumin from *Curcuma pseudomontana* produces AuNPs that have been used as antibacterial and anti-inflammatory agent. The synthesized AuNPs have been stable for up to five months^17^. Similarly, extracts from the leaves of *Populas alba*, *Lantum camara*, *Hibiscus arboreus*, and *M. oleifera* were utilized to synthesized stable AuNPs that shown potent antioxidant and antibacterial properties as well as the ability to photodegrade dyes^18,19^. The syntheses and stabilization of nanoparticles are facilitated by phytoconstituents, primarily flavonoids, according to earlier study^20^.

*E. diffusum D. is* the sole living representative of family Equisetaceae, native to Himalaya mountains and popularly referred to as the "Himalayan horse tail"^21^. *E. diffusum* is one of the most common medicinal plants with therapeutic qualities. *E. diffusum* D has been previously reported in the treatment of the bone fracture, kidney troubles^22^, diuretic effect^23^, bone dislocation^24^, fever, gonorrhea, urinary troubles, arthritis, as a cooling medicine^25^, scabies, skin disease^26^. The aim of the current study was to synthesize AuNPs using green synthesis approach. In addition, the green synthesized AuNPs were thoroughly characterized by different analytical and spectroscopic techniques and then evaluated the biological activities of the green synthesized AuNPs as antimicrobial, anticancer, antioxidant and antidiabetic agents.

## Materials and methods

### Materials

Samples of *Equisetum diffusum* were collected from the North Waziristan Tribal district, Khyber Pakhtunkhwa, Pakistan. A botanist (Ms. Naima Huma Naveed), Assistant Professor from the Department of Botany, University of Sargodha, Punjab, Pakistan, identified and authenticated the plant sample. The voucher number (SARGU/CHEM-027), was assigned to the plant specimen and then deposited to the herbarium (University of Sargodha-SARGU), Department of Botany, University of Sargodha, Punjab, Pakistan. HAuCl_4_ (Sigma-Aldrich®), DPPH (Sigma-Aldrich®), ethanol (Sigma-Aldrich®), and Mueller Hinton agar (Oxide) were procured from the local market. All chemicals were of analytical grade, and no additional purification was required. The solutions were prepared and the extractions were carried out using deionized water (DH_2_O). Bacterial strains were obtained from the Biochemistry Lab at, Institute of Chemistry, University of Sargodha, Sargodha, Pakistan.

### Extraction of aqueous extract

Freshly collected *E. diffusum* sample were thoroughly washed with tap and deionized water to remove any impurities. Then cut into small pieces, and shade-dried for 12 days, following a the method described by Assad et al.^27^. The plant material was finely ground into a superfine powder using a pestle and mortar. To prepare the plant powder suspension, 10 g of the powdered material were mixed with 200 mL of deionized water (DH2O) and stirred at room temperature for 5 h. The mixture was then filtered through Whatman No. 42 filter paper. The resulting filtrate was poured into a Petri dish and allowed to dry completely for 24 h at 45 °C in an air-drying oven. Once fully dried, the extract was carefully collected from the Petri dish and stored in an Eppendorf tube for later use.

### Green synthesis of AuNPs

**Fig. 1:**
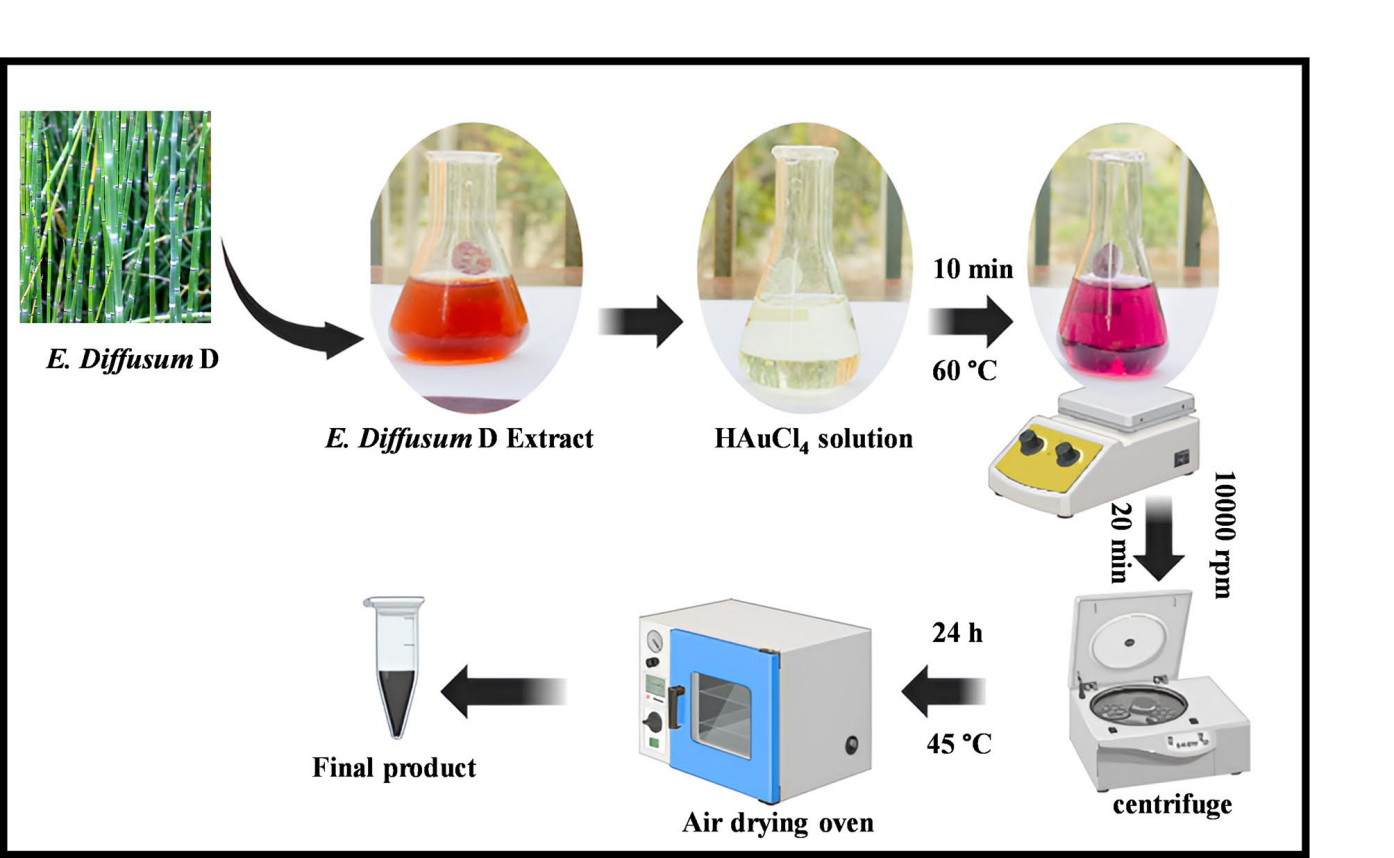
Preparation of AuNPs via green methodology.

For biosynthesis of AuNPs, 1 mM chloroauric acid (HAuCl_4_) was prepared by dissolving 33.97 mg in 100 mL DH_2_O. *E. diffusum* D extract and HAuCl_4_ solution were subjected to assessment in combination ratio 2:8, following the protocol of Geetha et al.^28^. The reaction mixture was stirrer for 10 min on hotplate at 60 °C. Within 10 min, the process of reducing gold ions to AuNPs was completed. As AuNPs develop the solution’s color changes from light yellow to ruby red. The synthesized AuNPs was centrifuge at 10,000 rpm for 20 min and then dried at 45 °C for 24 h as shown in Fig. 1. Then the dried sample was stored in Eppendorf tube and mentioned as ED@AuNPs. The decrease of metal ion concentration was carefully monitored using both visual examination and measurements by various analytical and spectroscopic techniques.

### Characterization

Biosynthesized ED@AuNPs was studied by recording their UV–vis absorption spectra at 300 to 800 nm using a Shimadzu UV-1800 spectrophotometer. Functional group starching as well as plant molecule functional groups on the particles surfaces were analyzed with, Fourier transform infrared spectrum (Shimadzu FTIR 8400S) in range of 4000–400 cm^−1^ region using the KBr pellets technique. The XRD analysis (JDX3532, JEOL, Tokyo, Japan) was used to analyzed ED@AuNPs phases identity and crystalline size. The scanning electron microscopy technique (Carbon Sticker No. G3347) Plano, (Wetzlar, Germany) was used to study the morphological aspects, and EDX was used to assess the elemental spectrum of ED@AuNPs. The interface charges were studied using zeta potential, and their size was evaluated using a zeta size distribution analyzer (Malvern Zetasizer Nano ZS).

### Antibacterial activity

For testing the antibacterial activity of biosynthesized ED@AuNPs, following methodology established by Afzal et al., (2024) with a few changes was employed^29^. Mueller Hinton agar (9.6 g, Oxoid) was dissolved in 160 mL of deionized water. Both the agar solution and Petri plates were sterilized in an autoclave at 121 °C for 20 min. The sterilized, MH agar medium was cooled to 50 °C, and about 25 mL was carefully poured into each Petri plate under aseptic conditions. The plates were then left at room temperature for 20 min to allow the agar to solidify before use. Pure strains were sub cultured on MH agar with in a rotating shaker set at 200 rpm at 37 °C. Following the streak plate procedure, the following bacterial strains were introduced into each Petri dish: *Staphylococcus epidermidis* (ATCC 12228), *Listeria monocytogenes* (ATCC 13932), *Staphylococcus aureus* (ATTC 9144)*, Pseudomonas aeruginosa (*ATCC 10145),* Bordetella bronchiseptica* (ATTC 4617), and *Escherichia coli strain (ATCC 10536)*. Five wells were created using a cork borer after spreading bacterial strains, and were labelled according to alphabetical order. The following sample were added to each well of the Petri dishes: (A) 30 µg/mL of ciprofloxacin (positive controls), (B) 20 µg/mL of ED@AuNPs, (C) 30 µg/mL ED@AuNPs, (D) 40 µg/mL of ED@AuNPs and (E) DH_2_O (negative control). The dishes were thereafter put in an incubator set at 37 °C for 24 h. The whole procedure was carried out under controlled laboratory settings using a laminar flow cabinet. Values were shown as the mean ± standard deviation and the antibacterial activity was tested in triplicate.

The minimum inhibitory concentration (MIC) of green-synthesized ED@AuNPs was assessed using the standard method outlined by the Clinical and Laboratory Standards Institute (CLSI)^30^. To assess the minimum inhibitory concentration (MIC) of ED@AuNPs, tested against six bacterial strains, including Gram-positive species (*S. epidermidis, L. monocytogenes, S. aureus*) and Gram-negative species (*P. aeruginosa, B. bronchiseptica, E. coli*). The bacterial strains were cultured in Mueller–Hinton (MH) broth at 37 °C with orbital agitation (200 rpm) for 24 h. Using optical density calibration, was standardized the inocula to a density of 1 × 10⁸ CFU/mL. To determine MIC values, the broth microdilution method, exposing bacteria to ED@AuNPs at concentrations ranging from 10 to 80 μg/mL was performed. For negative controls, nutrient broth supplemented with 80 μg/mL of ED@AuNPs, while positive controls contained only bacterial cultures in nutrient broth. Then incubated all test and control tubes at 37 °C for 24 h. After incubation, identified the MIC as the lowest ED@AuNPs concentration that completely prevented visible bacterial growth, based on optical clarity or turbidity measurements.

### Anticancer activity

#### Cell culture

Hepatic carcinomas (HePG2) cells were procured from the American Type Culture Collections Virginia(USA). The cells were cultured in DMEM supplemented with 10% FBS and 20 uL/mL of penicillin and streptomycin. The cultures were kept in a humidified environment with 5% CO_2_ for an incubation period of 37 °C.

#### MTT cytotoxic assay

For cell culture, 96 well plates were used to cultivate 1 × 106 HePG2 cells. Then subjected the cells to an overnight incubation period before introducing varying quantities of PPGE along with PPE (200, 150, 100, 50, 20, and 10 µg/mL). The + ive control group took taxol, whereas the negative control group used unpolished media. After 24 h, 48 h, and 72 h 20 µg/mL of the MTT solution was added to each well and let it incubate for 4 h. In order to get exact results, we created three separate wells. The absorbance was measured using an ELISA reader to measure absorbance at two different wavelengths, 540 nm and 690 nm, after 100 µL of DMSO was added. The ED@AuNPs cytotoxicity was evaluated by comparing the absorbing capacity of control and treated cells using the provided formula (Eq. 1):

1 $${\text{\% Cytotoxicity}} = \left( {\frac{{Absorbance \;of\; treated \;cells\left( {A1} \right)}}{{absorbance \;of \;negative \;control \left( {AO} \right) }}} \right) \times 100$$

### Antidiabetic activity

#### Starch hydrolysis

Starch hydrolysis was assess by observing the inhibitory zone on Petri plates, by following the method explained by Khan et al.^1^ with minimal modifications. On agar plates with 1.5% agar and 1% starch by weight, four holes were punched using a cork borer. As a control, well (A) was supplemented with an *Aspergillus oryzae* α-amylase mixture (EC 3.2.1.1) with 2 U/mL in a phosphate buffer solution (pH 6.9). In well B standard (Acarbose) (20, 30 µg/mL) and α-amylase, (C) ED@AuNPs (20, 30 µg/mL) and α-amylase and (D) ED extract (20, 30 µg/mL) and α-amylase, were mixed respectively. To quantify α-amylase activity, starch was stained using 0.5 mM I_2_ in 3% KI after 72 h incubation at 37 °C for 15 min. The hydrolyzed zone radius surrounding the wells was used to quantify the inhibitory results and measured in millimeter (mm). Results were presented as a percentage using (Eq. 2):

2 $$\% \; \alpha - amylase\; inhibition = \left( {\frac{diameter \;of\; control - diameter\; of\; sample)}{{diameter\; of\; the \;control }}} \right) \times 100$$

#### Enzyme kinetics

In order to conduct the amylase inhibition experiment, few adjustments to the standard operating procedure as stated in^31^ were employed. The mixture consisted of ED@AuNPs (ranging from 10 to 30 µg/mL) and 250 µg/L of amylase (0.4 U/mL) in a 0.02 M phosphate buffered solution (PBS) with a pH of 6.9 and 0.06 M sodium acetate. Adding 250 µL of starch solution 1% (w/v) in 0.02 M PBS with a pH of 6.9 and incubated for 10 min at 37 °C, and followed by another 30 min of incubation. After mixing 250 µL of dinitrosalicylic acid coloring reagent (DNS 96 mM, 30% Na–K tartrate, 0.4 M NaOH), the mixture was heated in a boiling water bath for 10 min to halt the reaction. After cooling to room temperature and diluted with to 2 mL PBS, absorbance was recorded at 540 nm.

The following formula was used to obtain the percentage of inhibition (Eq. 3):

3 $${\text{\% }}\;{\text{ inhibition}} = \left( {\frac{K - S)}{{K }}} \right) \times 100$$

K = Absorption of negative controls; S = Absorbance of sample/Absorbance of positive control.

### Antioxidant activity

#### FRAP assay

FRAP assay was carried out following the protocol outlined by Shobha et al.^32^ with minor modifications. A solution was prepared by mixing 2.5 mL of sodium phosphate buffer (0.2 M, pH 6.6) with 2.5 mL of 1% potassium ferricyanide. Then added varying concentrations of ED@AuNPs solution, ranging from 100 to 500 µg/mL. The mixture was incubated at 50 °C for 20 min. After incubation, added 10% (w/v) trichloroacetic acid and centrifuged the solution at 3000 rpm for 10 min. Then collected the supernatant and treated it with 2.5 mL of deionized water and 0.1% ferric chloride solution. Subsequently measured the absorbance of the solution at 700 nm using a Shimadzu UV-1800 spectrophotometer. The antioxidant potential of FRAP was calculated using the following equation (Eq. 4).

4 $$\text{X}=\left(\frac{y)}{0.0027 }\right) R2=0.9801$$

#### DPPH assay

Based on the approach described by Assad et al.^27^ briefly 1 mL methanolic DPPH (0.1 mM) solution of ED@AuNPs at concentrations ranging between 100 to 500 µg mL^−1^. After all the components were well mixed, the solutions were incubated at room temperature for 30 min in a dark chamber. A Shimadzu UV-1800 spectrophotometer with the wavelength set to 517 nm was used in order to ascertain the absorbance. Equation (Eq. 5) was used to determine the antioxidant potential of DPPH.

5 $${\text{\% RSA}} = \left( {\frac{Absorbance\; of\; control\; 517 - absorbance\; of \;sample \;517) }{{absorbance\; of \;control \;517}}} \right) \times 100$$

### Statistical analysis

Results were expressed as mean ± standard deviation. Origin Pro version 2021, and Office Excel 2016, were used for statistical analysis.

## Results and discussion

### Green synthesis of ED@AuNPs

**Fig. 2:**
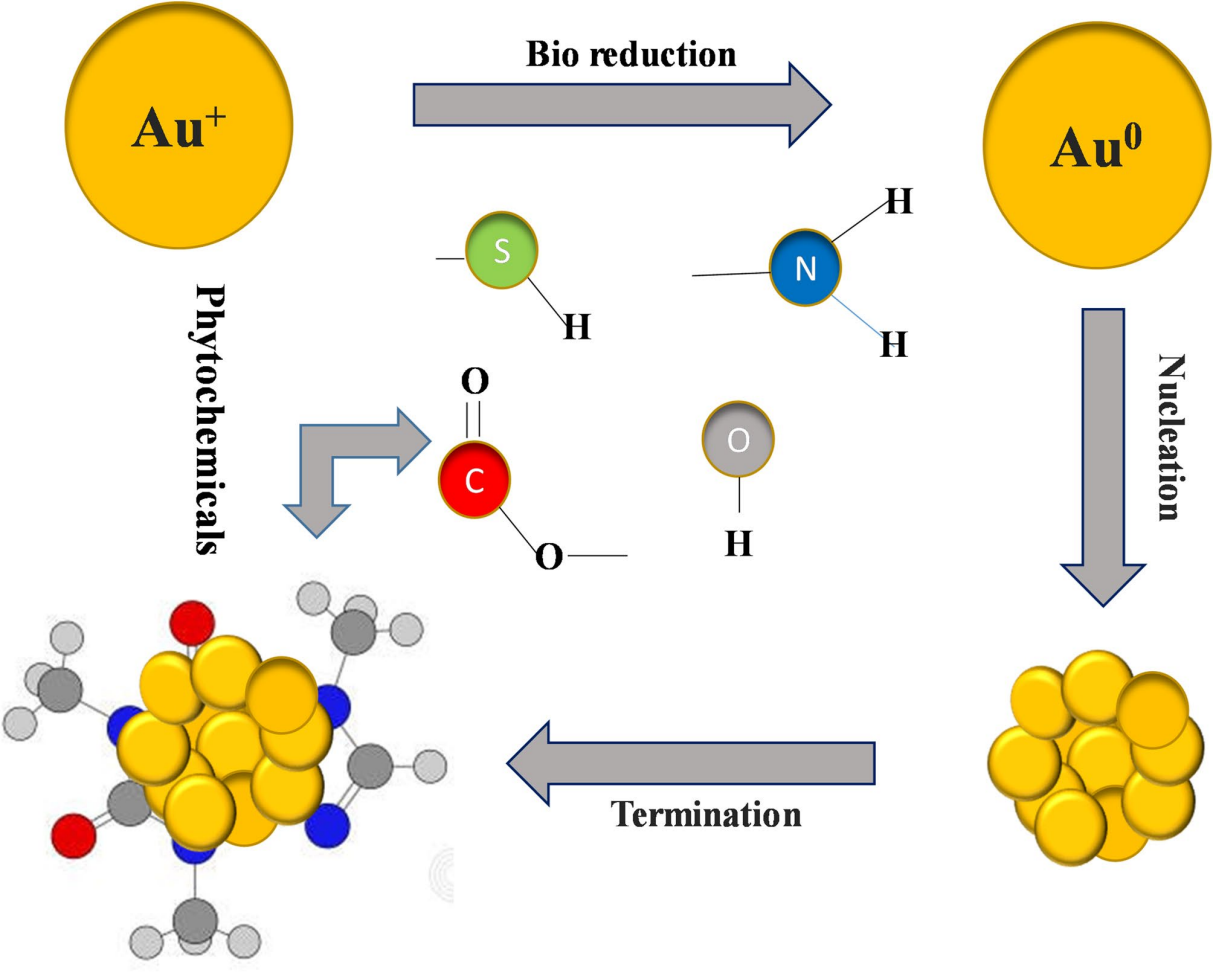
Mechanism of AuNPs green synthesis and phytochemicals involved in the reduction of Au ions.

The biosynthesis of AuNPs using *E. diffusum* extract involves a bioreduction process that is mediated through the phytochemicals of the plant^19^. The naturally occurring phytochemicals like flavonoids, terpenoids, and phenolic acids serve to act as reducing and stabilizing agents for the synthesis^27^. The mixing of extract of *E. diffusum* with chloroauric acid (HAuCl₄) solution, the phytochemicals reduce the gold ions (Au^3^⁺) by transferring electrons to them, converting into neutral gold atoms (Au⁰)^33^. The reaction is marked by a color change of the solution, typically ruby red or purple, as a sign of AuNP formation. The neutral gold atoms associate and create small clusters, which act as nucleation sites. As reduction proceeds, more Au atoms are deposited onto these clusters, and NPs growth occurs. The same phytochemicals that cause reduction also adsorb onto the surface of the newly formed AuNPs to form a capping layer. The capping layer prevents clumping of the NPs and renders them stable and well-dispersed in the solution. Mechanism of biosynthesis of AuNPs and reduction by phytochemicals are shown in Fig. 2.

### UV–vis spectroscopy of green synthesized ED@AuNPs

**Fig. 3:**
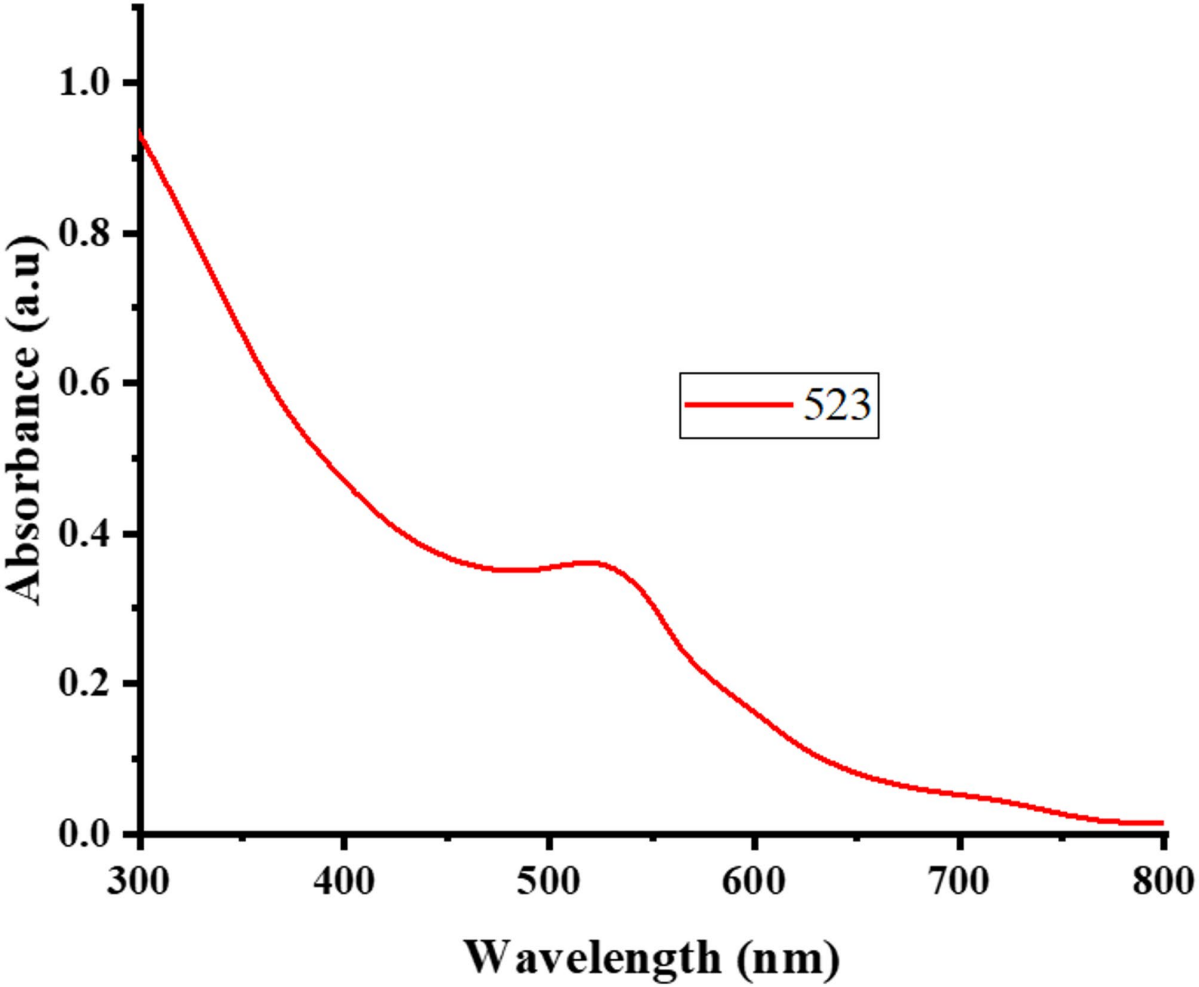
UV–vis spectroscopy of *E. diffusum* mediated green synthesized ED@AuNPs.

A distinctive peak at 523 nm was observed in the UV–Vis absorption spectra of green synthesized ED@AuNPs. This peak represents the surface Plasmon resonance (SPR)^19,34^. The presence of a peak at 523 nm in current investigation is indicative of the stable and uniformly sized gold nanoparticles formed as shown in Fig. 3. As the visible range is dominated by the SPR band of AuNPs, the observed diminishing intensity at larger wavelengths is in agreement with their expected behavior, indicating that the ED@AuNPs synthesis was effective and efficient. In previous study AuNPs were synthesized using *Ulva lactuca* L. extract from red algae, and a significant LSPR band was observed in the spectra at 529 nm^35^. In another study Botteon et al.^36^ observed LSPR band at 523 nm.

### XRD analysis

**Fig. 4:**
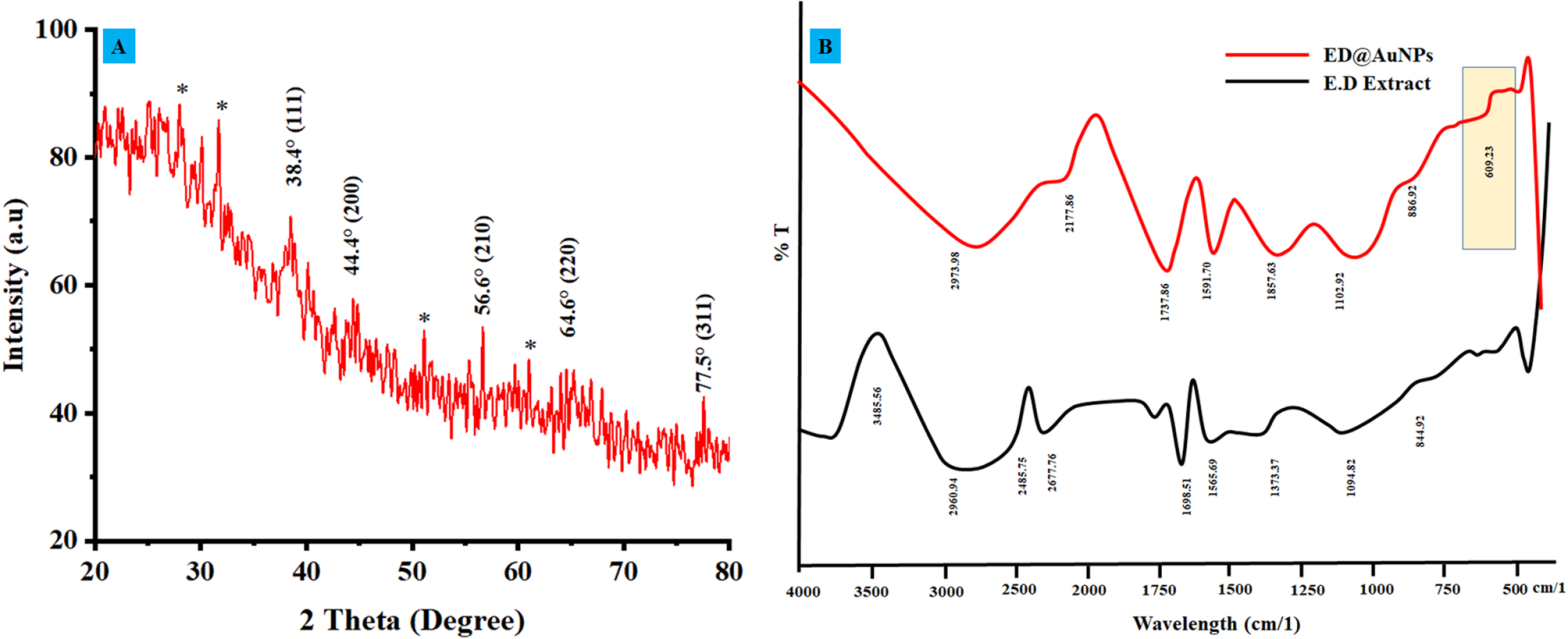
(**A**) Xrd analysis of green synthesized ED@AuNPs, (**B**) FTIR spectrum of *E. diffusum* D extract and green synthesized ED@AuNPs.

Different peaks of diffraction at 38.4°, 44.4°, 56.6°, 64.6°, as well as 77.5° were observed in the XRD pattern of the green synthesized ED@AuNPs as shown in Fig. 4A. These peaks corresponded roughly to the (111), (200), (210), (220), and (311) crystal structure planes of cubic face-centered (FCC) gold, respectively^37^. These distinctive peaks validate that the biosynthesized AuNPs are crystalline in structure. Possible secondary phases or contaminants, such as leftover plant biomolecules from the green synthesis process, were indicated by minor peaks with asterisk-marked. In general, the XRD pattern confirms that AuNPs possess high crystalline nature. Our results were in good agreement with previous reported literature^38^. The average crystallite size (42.66 nm) of the biosynthesized ED@AuNPs was calculated using Scherrer’s equation (Eq. 6)

6 $$D=\frac{k\lambda }{\beta Cos\theta }$$

where D = Crystal size (nm), K = Scherrer constant (dimensionless, typically 0.9), λ = X-ray wavelength (typically 1.5406 Å for Cu–Kα), β = Full width at half maximum (FWHM) of the diffraction peak (radians), θ = Bragg’s diffraction angle (in radians).

Dislocation density (δ) represents the number of dislocations present in a crystal structure per unit volume. The dislocation density (δ) of the biosynthesized ED@AuNPs is approximately 5.49 × 10^14^ lines/m^2^. Dislocation density (δ) was determined using the following equation (Eq. 7):

7 $$\updelta =\frac{1}{D2}$$

where δ = dislocation density (lines/m^2^), D = crystallite size (nm).

For ED@AuNPs, the calculated dislocation density (δ = 5.49 × 10^14^ lines/m^2^) suggests a moderately crystalline structure with some natural defects. This finding indicates that the biosynthesized NPs have high crystallinity, with minor imperfections likely resulting from the green synthesis process, such as residual biomolecules or lattice strain. The observed dislocation density reflects structural stability, making ED@AuNPs well-suited for applications in catalysis, biosensing, and biomedical fields, where controlled crystallinity is essential for optimal performance.

### FTIR analysis of biosynthesized ED@AuNPs

The FTIR spectra show a comparison of the plant extract (ED Extract) with the green synthesized ED@AuNPs as shown in Fig. 4B. Several noticeable peaks can be seen regarding the ED Extract spectrum. The first among these is a wide range at 3485.56 cm⁻^1^, which is related to O–H vibrations of stretching released by hydroxyl groups^39^. The other two peaks, at 2906.94 cm⁻^1^ alongside 1668.51 cm⁻^3^, are linked to C–H stretching vibrations and C=O stretching vibrations, respectively, released by carbonyl groups. The reduction and stabilization of the AuNPs are accomplished by these functional groups found in the plant extract. ED@AuNPs spectrum shows few changes and new peaks, like at 2937.38 cm⁻^3^ and 1102.92 cm⁻^3^. This shows that ED@AuNPs and phytochemicals from the plant extract are interacting with each other, as shown in Table 1. These changes show that the plant extract effectively stabilized and capped the ED@AuNPs. Furthermore, the signal at 609.23 cm⁻^1^ provides further evidence that ED@AuNPs were synthesized and stabilized using environmentally friendly processes, since it implies the establishment of Au-O bonds.

**Table 1:** FTIR analysis of ED@AuNPs and ED extract.

ED Extract	ED@AuNPs	Functional group
3485.56	–	O–H stretching (Hydroxyl group)
2906.94	2937.38	C–H stretching (Alkane)
1668.51	1737.86	C=O stretching (Carbonyl group)
1109.82	1102.92	C–O stretching (Alcohol)
844.92	886.92	C–H bending (Aromatic)
–	609.23	Metal–Oxygen bond (Au–O interaction)

### SEM analysis

**Fig. 5:**
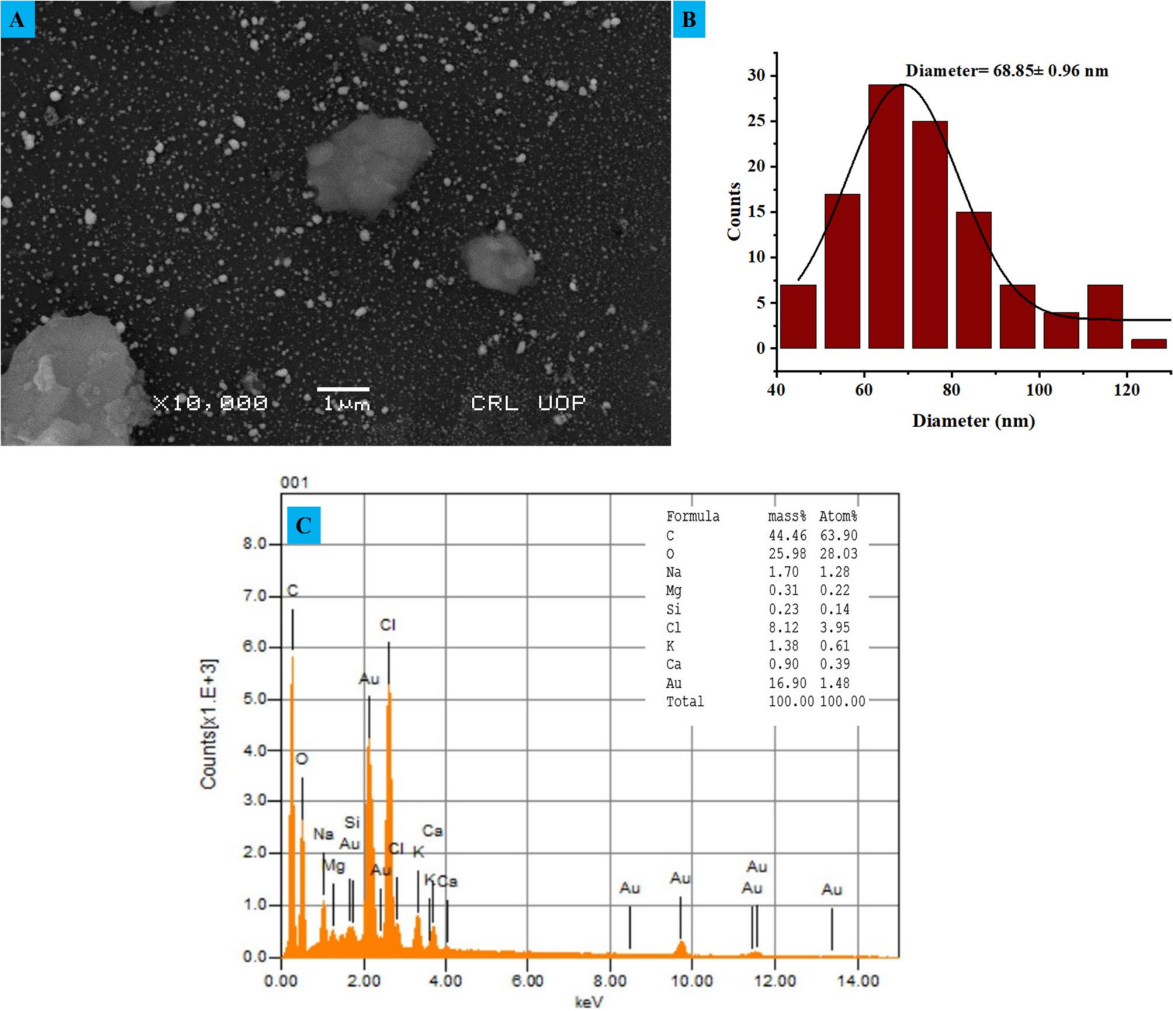
(**A**) SEM analysis of green synthesized ED@AuNPs, (**B**) average size distribution histogram of ED@AuNPs, (**C**) EDX spectra of green synthesize ED@AuNPs.

The size distribution histogram and scanning electron microscope (SEM) images of the green synthesized ED@AuNPs provide insight details about their shape and dimensions. The scanning electron microscopy (SEM) image at 10,000 × magnification reveals a population of NPs, that is evenly distributed and has very little aggregation as shown in Fig. 5A. According to the histogram, the ED@AuNPs typically vary in size from 60 to 80 nm (nm), and the average diameter is 68.85 ± 0.96 nm, as shown in Fig. 5B. The effectiveness of the biological molecules in plants extraction in limiting excessive particle development or aggregation is supported by the minute presence of bigger particles, which shows that they are effectively capped and stabilized. Results from scanning electron microscopy (SEM) show that green synthesis successfully produced stable, appropriately sized NPs. Ghafoor et al.^40^ study, pineapple extract for green synthesis of AuNPs. The NPs were nearly spherical in shape, with particle sizes ranging from 20 to 70 nm, as evident from the SEM analysis.

### EDX analysis

Elements present in the sample were confirmed by the EDX spectra of green synthsized ED@AuNPs as shown in Fig. 5C. The presence of noticeable peaks approximately 2.1 keV, 9.7 keV, and 11.4 keV for gold (Au) suggests that the production of ED@AuNPs was effective. Elements including silicon (Si), oxygen (O), carbon (C), and chlorine (Cl) are presumably derived from the plant extracts used in a green synthesis method, and peaks for these elements are also seen alongside gold. The existence of these extra components points to organic compounds that are stabilizing and encasing the ED@AuNPs. Potassium, sodium, and calcium found in the extract could be due to contaminants or lingering biomolecules. The production of ED@AuNPs and the role of biomolecules derived from plants in stabilizing them were both confirmed by the EDX study.

### Dynamic light scattering analysis of ED@AuNPs

**Fig. 6:**
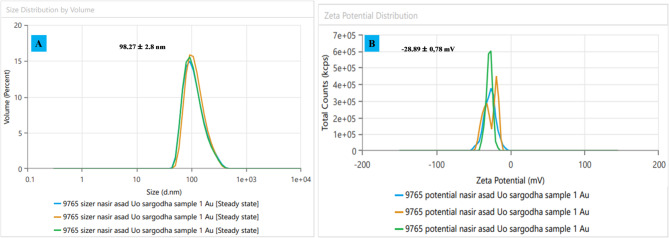
Dynamic light scattering analysis of green synthesized ED@AuNPs, (A) zeta sizer, (B) zeta potential.

The dynamic light scattering (DLS) data shown in Fig. 6A, show how the green manufactured ED@AuNPs are spread out in terms of size. The measurement of an average particle size of 98.27 ± 2.8 nm suggests that the sizes of the nanoparticles are distributed very uniformly with little fluctuation. Applications requiring consistent particle behavior would benefit greatly from the green synthesis method’s ability to generate nanoparticles with constant sizes, as shown by the narrow peak in the particle size distribution. The plant extract effectively caps and stabilizes the mixture, preventing bigger aggregates from forming, which is reflected in its consistency.

In Fig. 6B, the Zeta Potential Distribution measures the surface charge of the nanoparticles and provides insight into their stability in suspension. The Zeta potential of the gold nanoparticles is recorded as − 28.89 ± 0.78 mV, which indicates good colloidal stability. A Zeta potential with a high absolute value (either positive or negative) suggests that the nanoparticles are unlikely to aggregate due to electrostatic repulsion. The negative Zeta potential observed here suggests that the particles are well-dispersed and stable in solution, with a low likelihood of agglomeration, making them suitable for various biological and chemical applications.

### Antibacterial activity of green synthesized ED@AuNPs

**Fig. 7:**
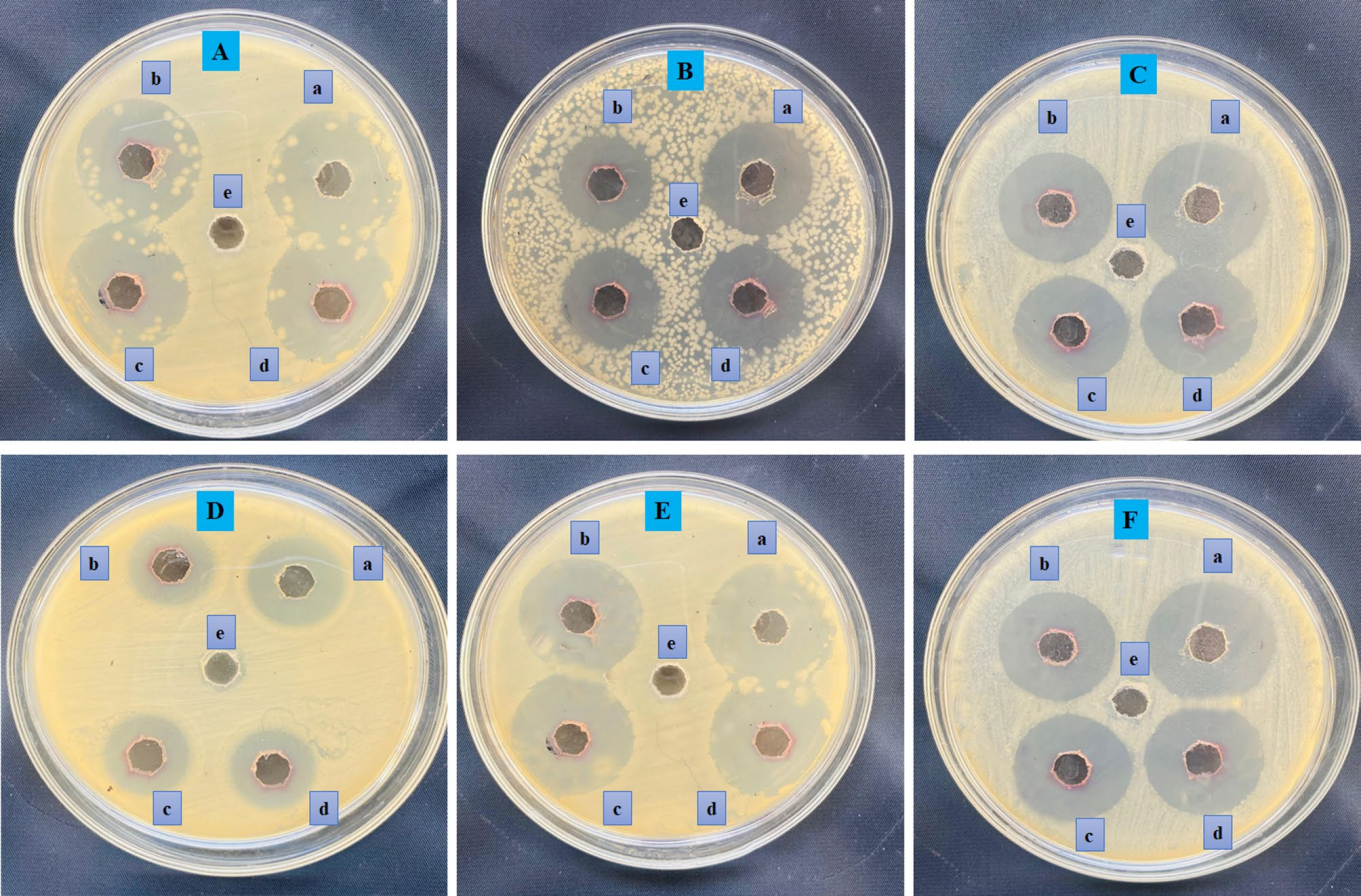
Antibacterial activity of green synthesize ED@AuNPs again both gram positive and gram negative bacterial strains, (**A**), *S. epidermidis* (**B**), *L. monocytogenes,* (**C**), *S. aureus* (**D**), *P. aeruginosa,* (**E**), *B. bronchiseptica* and (**F**), *E. coli*: (**a**) 30 µg/mL, positive control, (**b**) 20 µg/mL ED@AuNPs, (**c**) 30 µg/mL ED@AuNPs and (**d**) 40 µg/mL ED@AuNPs and (**e**) Negative control DH_2_O.

The misuse of antibiotics has become a prominent issue in recent years. Antibiotics released into the environment negatively impact bacteria in ecosystems, along with aquatic organisms, soil organisms, and plants. bacteria have the ability to become resistant to drugs, which may cause the rise of multi drug resistant (MDR) strains, often called "super bacteria." They can produce inactivating enzymes and hinder drugs by altering cytomembrane permeability^41^. Over 70% of bacteria currently demonstrate resistance to one or more antibiotics^42^. This necessitates an increase in antibiotic dosages by physicians, which may result in adverse reactions Fig. 7.

There is an urgent need to identify alternative materials to antibiotics for antibacterial applications. Research indicates that nanomaterial exhibit considerable antibacterial activity^43^. The elevated specific surface area enables nanoscale particles to attach significant amounts of functional ligands or serve as carriers for other active substances, thereby improving their interaction with target bacteria^44^.

**Fig. 8:**
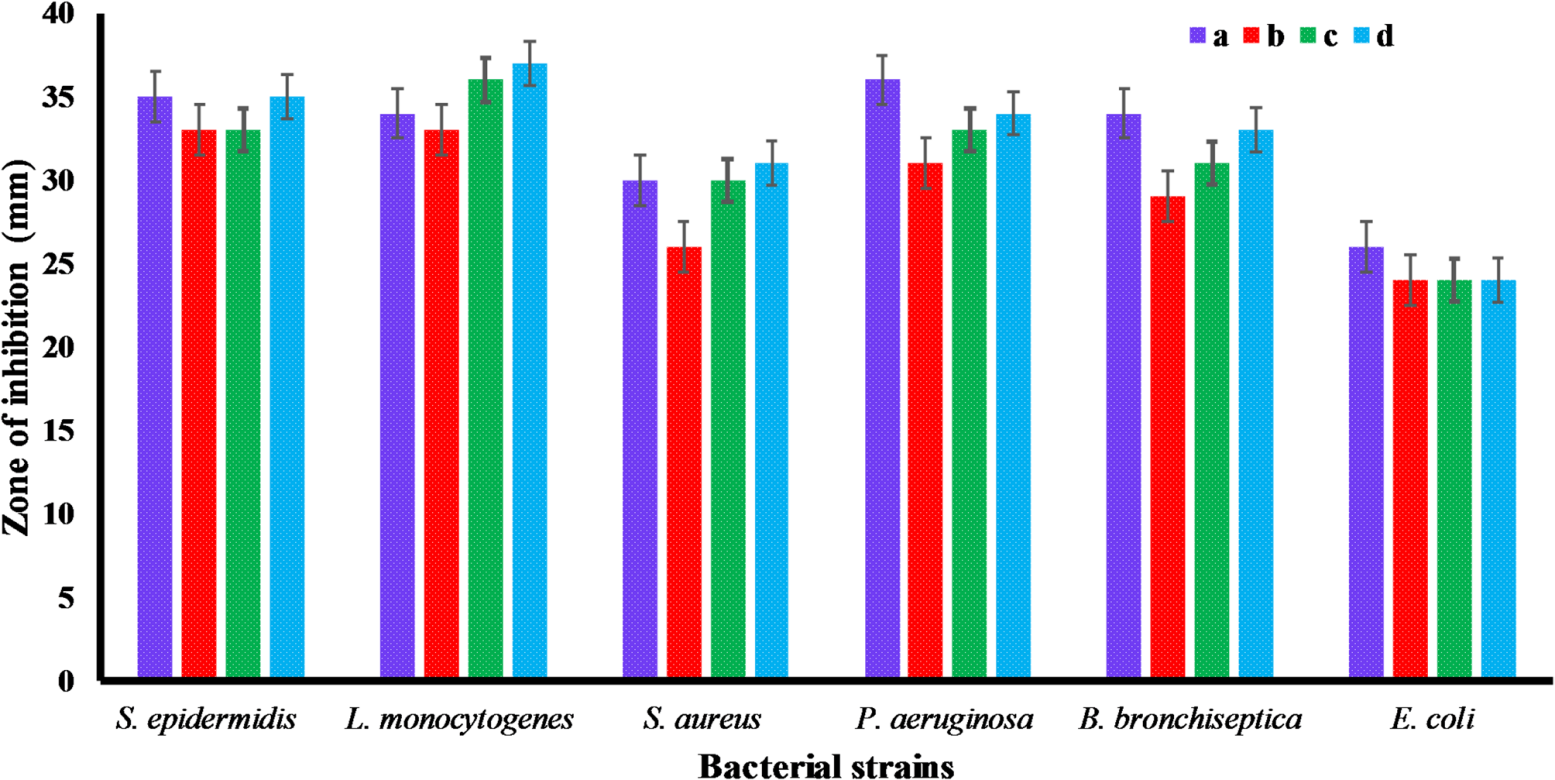
Graphical representation of antibacterial activity of green synthesize ED@AuNPs, (**a**) 30 µg/mL, positive control, (**b**) 20 µg/mL ED@AuNPs, (**c**) 30 µg/mL ED@AuNPs and (**d**) 40 µg/mL ED@AuNPs.

As demonstrated in Fig. 8, green synthesized gold nanoparticles (AuNPs) exhibited antibacterial properties. Both gram-positive and gram-negative bacteria were susceptible to the antibacterial effects of ED@AuNPs. Different concentrations of ED@AuNPs, shown by (a) (20 µg/mL) a positive control ciprofloxacin, (b) 20 µg/mL, (c) 30 µg/mL, (d) 40 µg/mL, ED@AuNPs and (e) negative control DH_2_O respectively, in each well, show that the ED@AuNPs have antibacterial activity. Larger zones surrounding the wells show higher antibacterial effects of the ED@AuNPs, whereas smaller zones show no inhibition of bacterial growth at all. The zones of inhibition against several bacterial strains, comprising *S. epidermidis L. monocytogenes, S. aureus P. aeruginosa, B. bronchiseptica* and *E. coli,* are measured in millimeters (mm) with respect to Fig. 7. Inhibition zones varied from approximately 24 to 35 mm throughout several strains and concentrations of AuNPs, as seen by the graph, demonstrating their strong antibacterial activities (Fig. 8). At 20 µg/mL, the zones of inhibition against *S. epidermidis*, *L. monocytogenes, S. aureus, P. aeruginosa, B. bronchiseptica* and *E. coli,* were 33, 33, 26, 31, 29, and 24 mm, respectively. Specifically, at a concentration of 30 µg/mL, the inhibition zones against *S. epidermidis L. monocytogenes, S. aureus, P. aeruginosa, B. bronchiseptica* and *E. coli* were 33, 36, 30, 33, 31, and 24 mm in that configuration. The zone of inhibition against *S. epidermidis* (35 mm), *L. monocytogenes* (37 mm), *S. aureus* (31 mm), *P. aeruginosa* (34 mm), *B. bronchiseptica* (33 mm), and *E. coli* (24 mm) at a concentration of 40 µg/mL. In compared to the positive control, erythromycin, our findings held up well across all concentrations. *E. coli* has the smallest inhibition zone, suggesting a relatively weak antibacterial response, in comparison with *S. epidermidis*, *L. monocytogenes* and *P. aeruginosa*, which all have bigger inhibition zones. Green produced AuNPs have a broad-spectrum antibacterial effect, as these studies show.

#### MIC of ED@AuNP*s*

The MICs of ED@AuNPs against the six bacterial species were estimated using the broth dilution method. The MIC was calculated by co-culturing bacteria for 24 h with different concentrations from 80 to 10 μg/mL, ED@AuNPs. The MIC is the lowest concentration at which the bacterial growth is completely inhibited. ED@AuNPs exhibited a MIC of 40 µg/mL against, S. *epidermidis*, 30 µg/mL against*, L. monocytogenes,* 50 µg/mL against, *S. aureus, P. aeruginosa, B. bronchiseptica* respectively and 60 µg/mL against*, E. coli*, (Table 2). Gram-positive bacteria showed greater susceptibility to ED@AuNPs compared to Gram-negative bacteria, as shown by the results of the antimicrobial susceptibility test. The size and zeta potential of AuNP*s* are important factors in their ability to penetrate the cell membrane and effectively killing the bacteria^45,46^. More specifically, the antibacterial activity of smaller ED@AuNPs is enhanced due to their smaller size and larger surface area compared to their larger counterparts. Moreover, the spherical shape of AuNPs is crucial for their absorption, which leads to cell death of microbes^47^.

**Table 2:** MIC (+ turbidity,—clarity) and ZOI of ED@AuNPs against gram positive and gram negative bacterial strains.

Bacterial strain	ED@AuNPs (µg/mL)								ZOI (mm)	MIC (µg/mL)
	10	20	30	40	50	60	70	80	40 µg/mL	
*S. epidermidis*	+	+	+	−	−	−	−	−	35 ± 1.3	40 ± 0.4
*L. monocytogenes*	+	+	−	−	−	−	−	−	37 ± 1.2	30 ± 0.3
*S. aureus,*	+	+	+	+	−	−	−	−	31 ± 1.2	50 ± 0.5
*P. aeruginosa*	+	+	+	+	−	−	−	−	33 ± 1.2	50 ± 0.2
*B. bronchiseptica*	+	+	+	+	−	−		−	34 ± 1.3	50 ± 0.2
*E. coli*	+	+	+	+	+	−	−	−	24 ± 1.4	60 ± 0.3

### Anticancer activity

The anti-cancer activity of ED@AuNPs was evaluated by the MTT cytotoxic assay against liver carcinoma (HePG2) cells cultured in DMEM medium supplemented with FBS and antibiotics. The assay measured cell viability after treatment with a series of concentrations of ED@AuNPs (10–200 µg/mL) for 24 h. The absorbance of treated and control cells was recorded at 540 nm and 690 nm, respectively, using an ELISA reader. The results expressed dose-dependent cytotoxicity of ED@AuNPs against HePG2 cells. At low concentrations (10 µg/mL), comparatively high cell viability (71.28%) was observed, indicating low cytotoxicity. It indicates that at low doses, the nanoparticles may have fewer influences on the metabolic activity of cells, in line with possible applications requiring biocompatibility. When the concentration is raised, however, the notable reduction in the cell viability was observed. At 20 µg/mL, viability was reduced to 66.30%, with subsequent reductions at 50 µg/mL (61.32%) and 100 µg/mL (56.20%). The greatest cytotoxicity was produced by the highest concentration examined, 200 µg/mL, reducing viability to 47.62% as shown in Table 3.

**Table 3:** Anticancer activity of ED@AuNPs.

Concentration µg/mL	Sample	Absorbance (1)	Absorbance (2)	Absorbance (3)	Viability (%) (1)	Viability (%) (2)	Viability (%) (3)	Average Viability (%)	S.D
40	Control	0.312	0.322	0.317	100.00	100.00	100.00	100.00	0.00
10	AuNPs	0.210	0.215	0.212	70.45	72.10	71.28	71.28	0.83
20	AuNPs	0.185	0.190	0.188	65.32	67.45	66.14	66.30	1.07
50	AuNPs	0.170	0.175	0.172	60.25	62.30	61.40	61.32	1.03
100	AuNPs	0.160	0.165	0.162	55.14	57.25	56.20	56.20	1.06
150	AuNPs	0.150	0.155	0.152	50.12	52.30	51.20	51.21	1.08
200	AuNPs	0.143	0.159	0.151	45.83	49.38	47.63	47.62	1.77

Increasing cytotoxicity with increased concentrations highlights the efficacy of ED@AuNPs in inducing metabolic stress in cancer cells, possibly through oxidative stress, membrane destabilization, or interference with cellular pathways. Reproducibility of results, as reflected by low standard deviations (for example, 0.83 at 10 µg/mL and 1.77 at 200 µg/mL), attests to the validity of the assay and reproducibility of results.

The results announce ED@AuNPs to be highly anticancer at elevated doses and can be perfect candidates for hepatic carcinoma therapeutics. Their dose-dependency suggests the potential to realize therapeutic effects through modulating their cytotoxicity to target selectively at desired locations, but with minimal damage to neighboring normal cells. Going forward, effort should be directed to understanding the molecular intricacies of this noted cytotoxicity and also exploring ED@AuNPs’ cancer cells vs. normal cell selectivity. Similar trends were reported by Paul et al.^48^.

### In vitro antidiabetic activity

#### Starch hydrolysis

Glucose intolerance as a result of inadequate insulin synthesis characterizes the metabolic disease family known as diabetes mellitus. Diabetes mellitus (DM) is thought to affect people all over the globe^49^. It may be caused by either a hereditary tendency or a learned malfunction of the pancreas, which in turn causes insulin resistance at receptors in the peripheral or a failure of insulin to carry out its intended function^50^. When insulin isn’t present, the metabolism of a diabetic gets out of hand, leading to an overabundance of carbohydrate, proteins, and fats. The most severe complications of diabetes mellitus include malfunction and failure of many organs as well as consistently high blood glucose levels. Diabetes mellitus (DM) is characterized by persistently high blood sugar levels, which raises microvascular and macro vascular risks of cardiovascular disease, hinders the metabolism of carbohydrates, lipids, and proteins, and causes prolonged damage that ultimately results in organ failure at sites like the renal system, eyes, nerve endings, heart, and vessel walls^51,52^. Recent estimates put the global diabetes population at about 150 million, with projections showing that figure rising to close to 300 million by the year 2025. over 8.5% of adults had a diabetes diagnosis in 2014, and the disease was a contributing factor in over 1.6 million fatalities in 2017^53^. Pakistan is one of the top ten nations in terms of the prevalence of diabetes, and it ranks seventh globally. Recent estimates put Pakistan’s diabetes prevalence at 7.6–11%; experts predict it will rise to 15% by 2030, putting the country in fourth place globally. Over 700 billion (or 10% more) is spent yearly on diabetes care worldwide, and popular drugs may have negative side effects if taken for too long^54^. Consequently, less invasive and more natural methods of preventing and treating diabetes are gaining popularity. There are several all-natural medications that can manage glucose metabolism and stop the deterioration of patients’ health, and they’re cheap and safe.

**Fig. 9:**
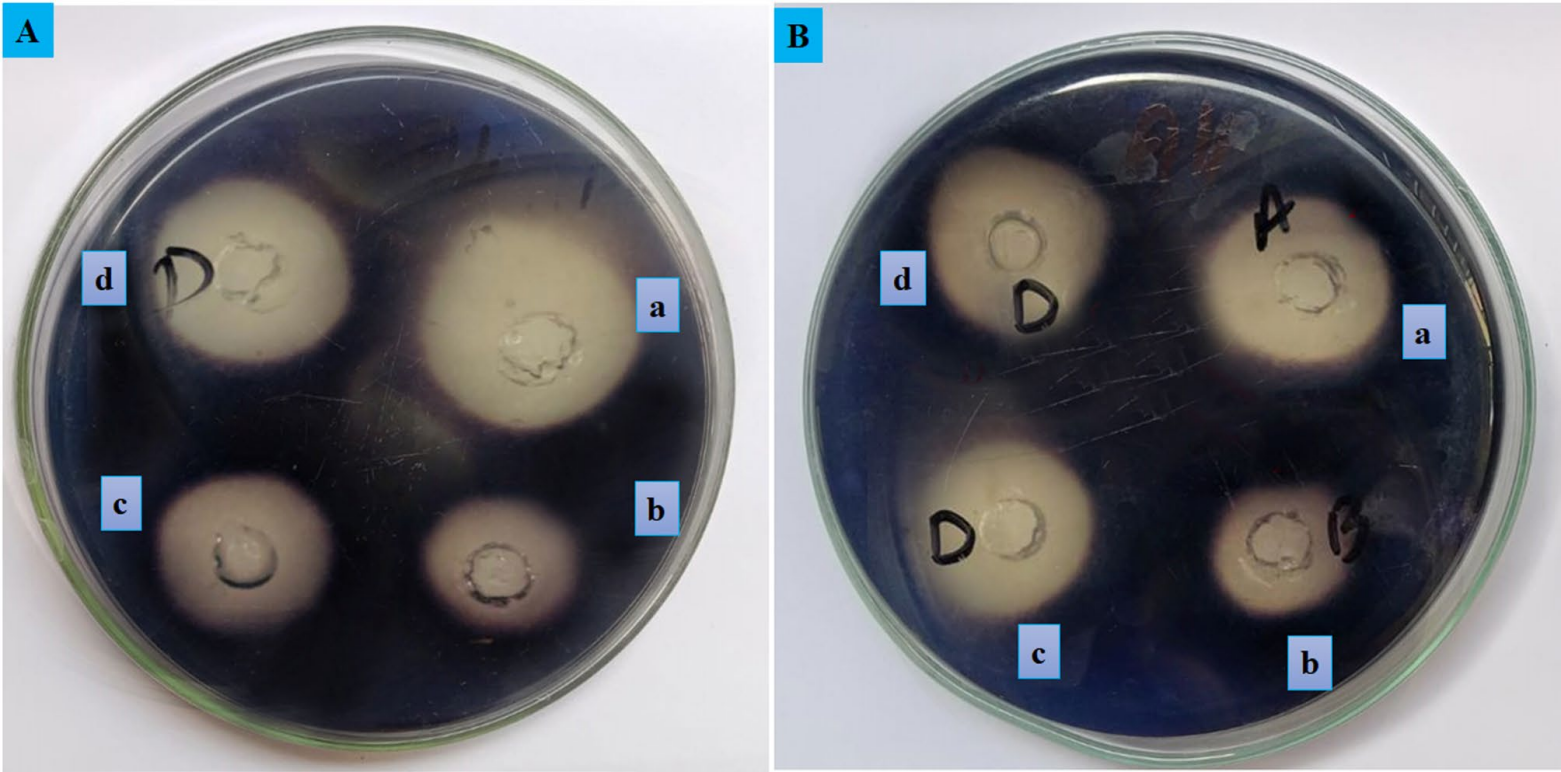
Inhibitory effect of ED@AuNPs on α-amylase enzyme using starch hydrolysis assay (**A**), (a) 20 µg/mL α-amylase enzyme only, (b) 20 µg/mL Standard Acarbose, (c) 20 µg/mL ED@AuNPs and (d) 20 µg/mL ED Extract and (**B**), (a) 30 µg/mL α-amylase enzyme only (b) 30 µg/mL Standard Acarbose, (**c**) 30 µg/mL ED@AuNPs and (d) 30 µg/mL ED Extract.

The smaller zone of inhibition indicates stronger enzyme inhibition, showing more effective antidiabetic action. The findings show that the antidiabetic effects of the AuNPs were higher than those of the plant extract and were comparable to the positive control, Acarbose, for two concentrations of green synthesized gold nanoparticles (ED@AuNPs) (20 µg/mL and 30 µg/mL, respectively). Figure 9A (20 µg/mL) shows that the AuNPs (well c) exhibited a zone of inhibition of 20 mm at 41.17% inhibition, which is superior to the 24 mm zone at 29.41% inhibition in the plant extract (well d) and the 18 mm zone at 47.05% inhibition in the positive control Acarbose (well b). Figure 9B (30 µg/mL) shows that the AuNPs (well c) had a 47.05% inhibition zone of 18 mm, which was greater than the plant extract (well d), 23.35% inhibition zone and comparable to the positive control (well b), 55.88% inhibition zone. The results demonstrate that the AuNPs outperform the plant extracts and comparable results to the control group in terms of antidiabetic action, with larger inhibition percentages that render them more efficient in preventing starch hydrolysis. % inhibition of starch hydrolysis is shown in Table 4.

**Table 4:** % inhibition of starch hydrolysis at concentration of 20 and 30 µg/mL of ED@AuNPs.

Solution	Concentration (µg/mL)	Zone of Inhibition (mm)	% Inhibition
Standard (Acarbose)	20	18	47.05
	30	15	55.88
ED@AuNPs	20	20	41.17
	30	18	47.05
ED extract	20	24	29.41
	30	23	32.35

#### Enzyme kinetic study

**Fig. 10:**
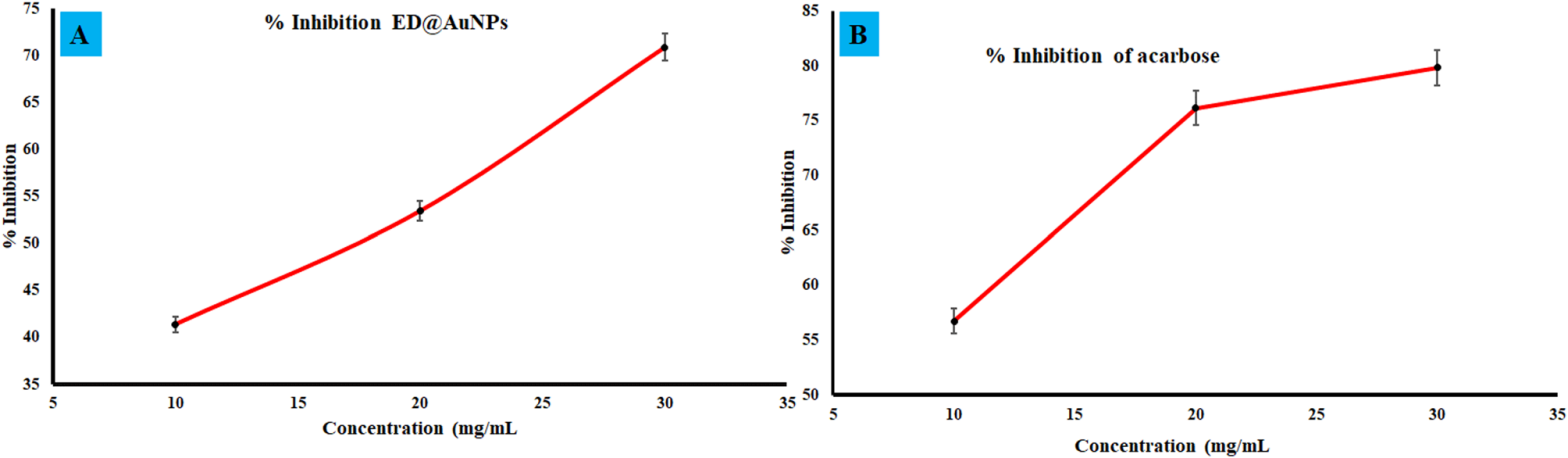
Enzyme kinetic study of green synthesized ED@AuNPs against α-amylase enzyme.

Depending on the concentration, AuNPs mediated through ED extract demonstrated significant α-amylase activity. At a dose of 30 µg/mL, the synthesized ED@AuNPs exhibited the highest inhibitory activity (70.85%) compared to 10 and 20 µg/mL, 41.39 and 53.42% respectively (Fig. 10A). Similarly, at a dosage of 30 µg/mL, the conventional medication Acarbose had the maximum inhibitory activity of 79.99% (Fig. 10B). The current research showed that ED extract mediated AuNPs showed strong α-amylase inhibitory action. Researchers looked at how antibiotics and antidiabetic medications interact with one another. It is hypothesized that ED@AuNPs also possess antidiabetic action, given their excellent antibacterial activity^1^.

### Antioxidant activity

**Fig. 11:**
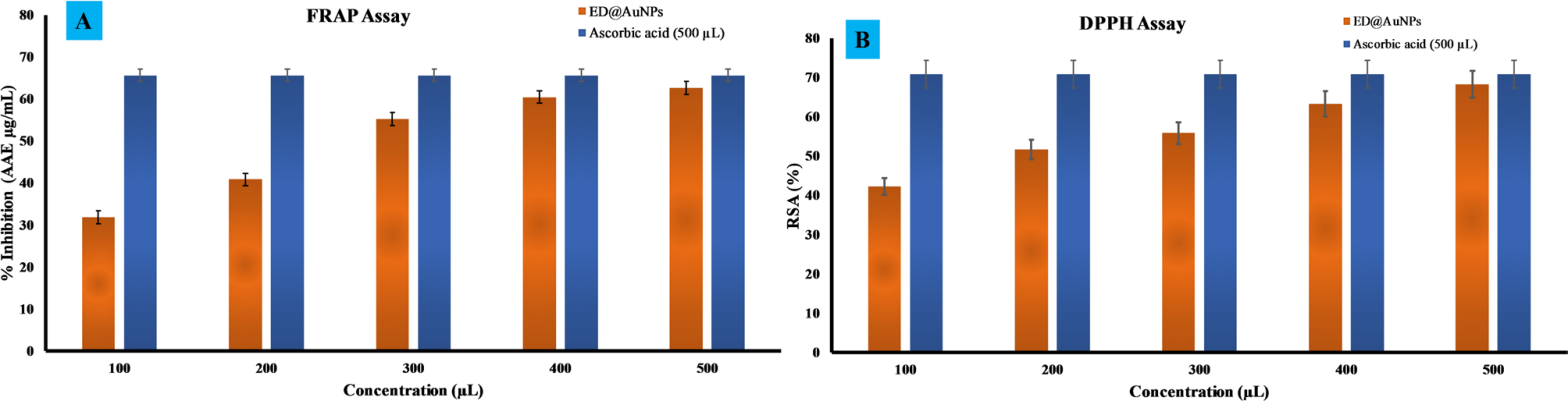
antioxidant activity of the green synthesized ED@AuNPs (**A**) FRAP assay and (**B**) DPPH assay.

Two different assays, the Ferric Reducing Antioxidant Power (FRAP) and the DPPH free radical scavenging test, were used to evaluate the antioxidant activity of biosynthesized ED@AuNPs. The biosynthesized ED@AuNPs showed concentration-dependent activity in the FRAP experiment (Fig. 11A), with values varying between 31.86 and 62.59 (AAE µg/mL). The antioxidant activity of the biosynthesized ED@AuNPs was comparable to that of the conventional antioxidant, ascorbic acid, at a concentration of 500 μL, indicating significant antioxidant activity. This indicates that the green-synthesized AuNPs show promise as antioxidants that may reduce ferric ions at greater concentrations. Figure 11B shows that the biosynthesized ED@AuNPs had a substantial free radical scavenging impact in the DPPH test, with values that range from 42.09 to 68.24 (%RSA). At 500 μg/mL, the activity was almost the same as the conventional ascorbic acid. This suggests that the AuNPs have great antioxidant properties, as they can successfully neutralize free radicals at larger concentrations. These findings demonstrate that green-synthesized AuNPs are effective antioxidants, particularly at high concentrations, and point to a wide range of possible uses for these NPs.

### Compression with previous literature

The Table 5 presents a comparison of diverse plant and algae sources, their particle sizes, and related biological activity. The sources vary from *P. minima* (15 nm), recognized for anti-diabetic and antibacterial capabilities, to *E. diffusum* (69 nm), which is being studied for medicinal purposes. Most sources demonstrate antibacterial action, with particle diameters range from 10 nm in *C. ternatea* to 100 nm in *A. calamus*. *B. propolis* (15 nm) stands out for its combination of antibacterial and anticancer properties. Furthermore, some sources such as *B. hispida* (23 nm) are examined for in vitro toxicity. This comparison demonstrates the varied bioactivities associated with different NPs sizes, providing insights into possible uses in health and therapy.

**Table 5:** Compression of biosynthesized ED@AuNPs with previous literature.

S. No.	Source	Part used	Size (nm)	Activities	References
1	*P. minima*	Whole plant	15	Anti-diabetic, anti-microbial activities	^55^
2	*G. elongata*	Whole algae	77	Antibacterial activity	^56^
3	*A. calamus*	Rhizomes	100	Antibacterial activity	^57^
4	*A. comosus*	Leaves	16	Antibacterial activity	^58^
5	*B. hispida*	Peel	23	In vitro toxicity	^16^
6	*B. propolis*	Bee propolis	15	Antimicrobial, anticancer activities	^36^
7	*C. ternatea*	Leaves	10	Antibacterial activity	^59^
8	*A. paeoniifolius*	Tuber	22	Antibacterial activity	^60^
10	*J. integerrima*	Flower	39	Antibacterial activity	^61^
11	*E. Diffusum*	Whole plant	69	Medicinal application	This work

## Conclusions

This study successfully demonstrates the eco-friendly synthesis of gold nanoparticles (ED@AuNPs) using *Equisetum diffusum* a medicinal plant rich in phytochemicals that served as natural reducing and stabilizing agents. The nanoparticles, with a uniform size distribution of 60–80 nm and high stability, were thoroughly characterized using UV–vis spectroscopy, XRD, FTIR, SEM, EDX, and dynamic light scattering (DLS) analysis. The DLS analysis revealed that the synthesized nanoparticles had an average particle size of 98.27 ± 2.8 nm, indicating a narrow size distribution and minimal aggregation. The zeta potential of ED@AuNPs was recorded as -28.89 ± 0.78 mV, signifying excellent colloidal stability due to strong electrostatic repulsion, which prevents nanoparticle aggregation. The biological evaluations revealed significant multifunctional activities. ED@AuNPs exhibited broad-spectrum antibacterial activity against both Gram-positive and Gram-negative bacteria, with inhibition zones ranging from 24 to 37 mm, showing the strongest activity at 40 µg/mL. The anticancer potential of the nanoparticles was evident from the MTT assay against hepatic carcinoma (HePG2) cells, showing dose-dependent cytotoxicity. At low concentrations (10 µg/mL), cell viability remained high at 71.28%, while at the highest concentration (200 µg/mL), it reduced to 47.62%, indicating strong anticancer activity. Furthermore, ED@AuNPs demonstrated notable antidiabetic activity, with α-amylase inhibitory rates reaching up to 70.85% at 30 µg/mL, comparable to the standard drug Acarbose. The nanoparticles also exhibited robust antioxidant properties, as evidenced by FRAP and DPPH assays, where maximum reduction and free radical scavenging activities were achieved at 500 µg/mL, comparable to ascorbic acid. These results highlight the potential of ED@AuNPs as effective agents for biomedical applications, including antibacterial, anticancer, antidiabetic, and antioxidant therapies. The green synthesis approach provides a sustainable and cost-effective alternative to conventional methods, eliminating hazardous chemicals and reducing environmental impact. Future studies should focus on elucidating the molecular mechanisms of these activities and validating there in vivo efficacy to further explore their therapeutic potential.

## Data Availability

The data that support the findings of this study are available from the corresponding author upon reasonable request.

## References

[1] Khan, Z. U. R. et al. *Aconitum lycoctonum* L. (Ranunculaceae) mediated biogenic synthesis of silver nanoparticles as potential antioxidant, anti-inflammatory, antimicrobial and antidiabetic agents. *BMC Chem.* **17**, 128 (2023). 37770921 10.1186/s13065-023-01047-5 PMC10540474

[2] Hashmi, S. S. et al. Green synthesis of silver nanoparticles from *Olea europaea* L. extracted polysaccharides, characterization, and its assessment as an antimicrobial agent against multiple pathogenic microbes. *Open Chem.* **22**, 20240016 (2024).

[3] Noor, A. et al. Green synthesis of silver-doped ZnO nanoparticles From *Adiantum venustum* D. Don (Pteridaceae): Antimicrobial and antioxidant evaluation. *J. Basic Microbiol.* **65**, e2400543 (2025). 39807572 10.1002/jobm.202400543

[4] Ghaffar, S. et al. Improved photocatalytic and antioxidant activity of olive fruit extract-mediated ZnO nanoparticles. *Antioxidants* **12**, 1201 (2023). 37371931 10.3390/antiox12061201 PMC10295640

[5] Jabbar, A. et al. A highly selective Hg 2+ colorimetric sensor and antimicrobial agent based on green synthesized silver nanoparticles using Equisetum diffusum extract. *RSC Adv.* **13**, 28666–28675 (2023). 37790097 10.1039/d3ra05070j PMC10543206

[6] Ray, P. C. Size and shape dependent second order nonlinear optical properties of nanomaterials and their application in biological and chemical sensing. *Chem. Rev.* **110**, 5332–5365 (2010). 20469927 10.1021/cr900335q PMC2935945

[7] Bhardwaj, K. et al. Biogenic metallic nanoparticles from seed extracts: Characteristics, properties, and applications. *J. Nanomater.* **2022**, 2271278 (2022).

[8] Khan, S. A., Shahid, S. & Lee, C.-S. Green synthesis of gold and silver nanoparticles using leaf extract of *Clerodendrum inerme*; characterization, antimicrobial, and antioxidant activities. *Biomolecules* **10**, 835 (2020). 32486004 10.3390/biom10060835 PMC7356939

[9] Vundela, S. R. et al. Multi-biofunctional properties of phytofabricated selenium nanoparticles from *Carica papaya* fruit extract: Antioxidant, antimicrobial, antimycotoxin, anticancer, and biocompatibility. *Front. Microbiol.* **12**, 769891 (2022). 35250900 10.3389/fmicb.2021.769891 PMC8892101

[10] Ying, S. et al. Green synthesis of nanoparticles: Current developments and limitations. *Environ. Technol. Innov.* **26**, 102336 (2022).

[11] Alsammarraie, F. K., Wang, W., Zhou, P., Mustapha, A. & Lin, M. Green synthesis of silver nanoparticles using turmeric extracts and investigation of their antibacterial activities. *Colloids Surf. B* **171**, 398–405 (2018). 10.1016/j.colsurfb.2018.07.059 30071481

[12] Vijayaram, S. et al. Applications of green synthesized metal nanoparticles—A review. *Biol. Trace Elem. Res.* **202**, 360–386 (2024). 37046039 10.1007/s12011-023-03645-9 PMC10097525

[13] Hussain, S. B. et al. Characterization and comparative antibacterial activities of gold nanoparticles synthesized by *Tagetes patula* L. (Asteraceae) flower extract. *Microscopy Res. Tech.* **88**, 1869–1880 (2025). 10.1002/jemt.24829 39995015

[14] Lee, K. X. et al. Recent developments in the facile bio-synthesis of gold nanoparticles (AuNPs) and their biomedical applications. *IJN.* **15**, 275–300 (2020). 32021180 10.2147/IJN.S233789 PMC6970630

[15] Muddapur, U. M. et al. Plant-based synthesis of gold nanoparticles and theranostic applications: A review. *Molecules* **27**, 1391 (2022). 35209180 10.3390/molecules27041391 PMC8875495

[16] Al Saqr, A. et al. Synthesis of gold nanoparticles by using green machinery: Characterization and in vitro toxicity. *Nanomaterials* **11**, 808 (2021). 33809859 10.3390/nano11030808 PMC8004202

[17] Muniyappan, N., Pandeeswaran, M. & Amalraj, A. Green synthesis of gold nanoparticles using *Curcuma pseudomontana* isolated curcumin: Its characterization, antimicrobial, antioxidant and anti-inflammatory activities. *Environ. Chem. Ecotoxicol.* **3**, 117–124 (2021).

[18] Guliani, A., Kumari, A. & Acharya, A. Green synthesis of gold nanoparticles using aqueous leaf extract of *Populus alba*: Characterization, antibacterial and dye degradation activity. *Int. J. Environ. Sci. Technol.* **18**, 4007–4018 (2021).

[19] Boruah, J. S. et al. Green synthesis of gold nanoparticles using an antiepileptic plant extract: In vitro biological and photo-catalytic activities. *RSC Adv.* **11**, 28029–28041 (2021). 35480751 10.1039/d1ra02669k PMC9038048

[20] Assad, N. et al. Diffused sunlight assisted green synthesis of silver nanoparticles using *Cotoneaster nummularia* polar extract for antimicrobial and wound healing applications. *Nat. Prod. Res.* **39**, 2203–2217 (2023). 38146228 10.1080/14786419.2023.2295936

[21] Takuli, P., Khulbe, K., Kumar, P. & Pant, C. Chemical composition of essential oil of *Equisetum diffusum*: A noble source of phytol. *IJPSR.* **11**, 5572–5578 (2020).

[22] Singh, B. P. & Upadhyay, R. Medicinal pteridophytes of Madhya Pradesh. *J. Pharmacogn. Phytochem.* **3**, 173–176 (2014).

[23] Jain, S. K. *Dictionary of Indian Folk Medicine and Ethnobotany* (Deep publications, 1991).

[24] Sureshkumar, J. et al. A review on ethnomedicinally important pteridophytes of India. *J. Ethnopharmacol.* **219**, 269–287 (2018). 29578072 10.1016/j.jep.2018.03.024

[25] Chandra, S. & Srivastava, M. (eds) *Pteridology in the New Millennium* (Springer, 2003).

[26] Jha, P. K., Karmacharya, S. B., Chettri, M. K., Thapa, C. B. & Shrestha, B. B. *Medicinal Plants in Nepal-An Anthology of Contemporary Research* (Ecological society, 2008).

[27] Assad, N., Abbas, A., ur Rehman, M. F. & Naeem-ul-Hassan, M. Photo-catalytic and biological applications of phyto-functionalized zinc oxide nanoparticles synthesized using a polar extract of Equisetum diffusum D. *RSC Adv.* **14**, 22344–22358 (2024). 39010906 10.1039/d4ra03573a PMC11247436

[28] Geetha, R. et al. Green synthesis of gold nanoparticles and their anticancer activity. *Cancer Nano.* **4**, 91–98 (2013). 10.1007/s12645-013-0040-9 PMC4451866 26069504

[29] Afzal, M. et al. Photocatalytic degradation of organic dyes and biological potentials of biogenic zinc oxide nanoparticles synthesized using the polar extract of *Cyperus scariosus* R.Br. (Cyperaceae). *Green Process. Synth.* **13**, 20240038 (2024).

[30] Hsueh, P.-R. et al. Consensus statement on the adherence to clinical and laboratory standards institute (CLSI) antimicrobial susceptibility testing guidelines (CLSI-2010 and CLSI-2010-update) for enterobacteriaceae in clinical microbiology laboratories in Taiwan. *J. Microbiol. Immunol. Infect.* **43**, 452–455 (2010). 21075714 10.1016/S1684-1182(10)60070-9

[31] Wulandari, L., Pratoko, D. K., Khairunnisa, P. & Muyasaroh, L. Determination α-amylase inhibitor activity of methanol extract of coffee leaves using UV-Vis spectrophotometric method and validation. *IOP Conf. Ser. Earth Environ. Sci.* **743**, 012094 (2021).

[32] Shobha, N. et al. Synthesis and characterization of Zinc oxide nanoparticles utilizing seed source of *Ricinus communis* and study of its antioxidant, antifungal and anticancer activity. *Mater. Sci. Eng. C* **97**, 842–850 (2019). 10.1016/j.msec.2018.12.023 30678976

[33] Ahmad, S. et al. Green synthesis of gold and silver nanoparticles using crude extract of *Aconitum violaceum* and evaluation of their antibacterial, antioxidant and photocatalytic activities. *Front. Bioeng. Biotechnol.* **11**, 1320739 (2024). 38268939 10.3389/fbioe.2023.1320739 PMC10807692

[34] Tang, Y., Zeng, X. & Liang, J. Surface plasmon resonance: An introduction to a surface spectroscopy technique. *J. Chem. Educ.* **87**, 742–746 (2010). 21359107 10.1021/ed100186y PMC3045209

[35] González-Ballesteros, N. et al. Macroalgae to nanoparticles: Study of *Ulva lactuca* L. role in biosynthesis of gold and silver nanoparticles and of their cytotoxicity on colon cancer cell lines. *Mater. Sci. Eng. C.* **97**, 498–509 (2019). 10.1016/j.msec.2018.12.066 30678937

[36] Botteon, C. E. A. et al. Biosynthesis and characterization of gold nanoparticles using Brazilian red propolis and evaluation of its antimicrobial and anticancer activities. *Sci. Rep.* **11**, 1974 (2021). 33479338 10.1038/s41598-021-81281-w PMC7820602

[37] Ghoreishi, S. M. & Mortazavi-Derazkola, S. Eco-friendly synthesis of gold nanoparticles via tangerine peel extract: Unveiling their multifaceted biological and catalytic potentials. *Heliyon.* **11**, e40104 (2025). 39801986 10.1016/j.heliyon.2024.e40104 PMC11719353

[38] Shirzadi-Ahodashti, M. et al. Optimization and evaluation of anticancer, antifungal, catalytic, and antibacterial activities: Biosynthesis of spherical-shaped gold nanoparticles using Pistacia vera hull extract (AuNPs@ PV). *Arab. J. Chem.* **16**, 104423 (2023).

[39] Barzegarparay, F. et al. Green synthesis of novel selenium nanoparticles using *Crataegus monogyna* extract (SeNPs@ CM) and investigation of its toxicity, antioxidant capacity, and anticancer activity against MCF-7 as a breast cancer cell line. *Biomass Conv. Bioref.* **14**, 25369–25378 (2024).

[40] Abdul Ghafoor, D., Saod, W. M. & Mohammed, N. Green synthesis of gold nanoparticles using pineapple extract and study their analytical characterization and antibacterial activity. *Syst. Rev. Pharm* **11**(2), 462–465 (2020).

[41] Hu, Y. et al. Removal of sulfonamide antibiotic resistant bacterial and intracellular antibiotic resistance genes by UVC-activated peroxymonosulfate. *Chem. Eng. J.* **368**, 888–895 (2019).

[42] Allahverdiyev, A. M., Abamor, E. S., Bagirova, M. & Rafailovich, M. Antimicrobial effects of TiO_2_ and Ag_2_ O nanoparticles against drug-resistant bacteria and *Leishmania* parasites. *Fut. Microbiol.* **6**, 933–940 (2011). 10.2217/fmb.11.78 21861623

[43] Bouttier-Figueroa, D. C. et al. Green synthesis of gold nanoparticles via *Moringa oleifera* seed extract: antioxidant, antibacterial and anticarcinogenic activity on lung cancer. *J Environ. Sci. Health Part A* **59**, 231–240 (2024). 10.1080/10934529.2024.2366736 38881214

[44] Gupta, P. C., Sharma, N., Mishra, P., Rai, S. & Verma, T. Role of gold nanoparticles for targeted drug delivery. In *Metal and metal-oxide based nanomaterials* (eds Bachheti, R. K. et al.) 243–269 (Springer, 2024).

[45] Yousaf, H., Mehmood, A., Ahmad, K. S. & Raffi, M. Green synthesis of silver nanoparticles and their applications as an alternative antibacterial and antioxidant agents. *Mater. Sci. Eng., C* **112**, 110901 (2020). 10.1016/j.msec.2020.110901 32409057

[46] Geremew, A. et al. Green synthesis of novel silver nanoparticles using *Salvia blepharophylla* and *Salvia greggii*: Antioxidant and antidiabetic potential and effect on foodborne bacterial pathogens. *Int. J. Mol. Sci.* **25**, 904 (2024). 38255978 10.3390/ijms25020904 PMC10815671

[47] Ghaffar, N. et al. Metal nanoparticles assisted revival of Streptomycin against MDRS Staphylococcus aureus. *PLoS ONE* **17**, e0264588 (2022). 35324924 10.1371/journal.pone.0264588 PMC8947119

[48] Paul, P. et al. Thionine conjugated gold nanoparticles trigger apoptotic activity toward HepG2 cancer cell line. *ACS Biomater. Sci. Eng.* **4**, 635–646 (2018). 33418752 10.1021/acsbiomaterials.7b00390

[49] Batool, S. et al. Delving the role of the ameliorative effects of *Caralluma tuberculata* NE Br. (Apocynaceae) on diabetes and its effect on the organs weight of alloxan-induced adult male mice. *Pol. Environ. Stud.* **33**, 523–531 (2024).

[50] Rasool, S. et al. Toxicological effects of the chemical and green ZnO NPs on *Cyprinus carpio* L. observed under light and scanning electron microscopy. *Microscopy Res. Tech.* **85**, 848–860 (2022). 10.1002/jemt.23954 34655129

[51] Ramachandran, S. et al. Investigation of antidiabetic, antihyperlipidemic, and in vivo antioxidant properties of *Sphaeranthus indicus* Linn. In Type 1 diabetic rats: An Identification of possible biomarkers. *Evid. Based Complement. Altern. Med.* **2011**, 571721 (2011). 10.1155/2011/571721 PMC2952313 20953435

[52] Ozturk, M. et al. Potential medicinal plants used in the hypertension in Turkey, Pakistan, and Malaysia. In *Plant and Human Health* Vol. 1 (eds Ozturk, M. & Hakeem, K. R.) 595–618 (Springer, 2018).

[53] Organization WH. World Health Statistics 2016 [OP]: Monitoring Health for the Sustainable Development Goals (SDGs). (World Health Organization, 2016).

[54] Salleh, N. H. et al. Systematic review of medicinal plants used for treatment of diabetes in human clinical trials: An ASEAN perspective. *Evid. Based Complement Altern. Med.* **2021**, 1–10 (2021). 10.1155/2021/5570939 PMC8528580 34691218

[55] Sekar, V. et al. Synthesis of gold nanoparticles (AuNPs) with improved anti-diabetic, antioxidant and anti-microbial activity from *Physalis minima*. *J. King Saud Univ. Sci.* **34**, 102197 (2022).

[56] Abdel-Raouf, N., Al-Enazi, N. M. & Ibraheem, I. B. Green biosynthesis of gold nanoparticles using *Galaxaura elongata* and characterization of their antibacterial activity. *Arab. J. Chem.* **10**, S3029–S3039 (2017).

[57] Ganesan, R. M. & Prabu, H. G. Synthesis of gold nanoparticles using herbal *Acorus calamus* rhizome extract and coating on cotton fabric for antibacterial and UV blocking applications. *Arab. J. Chem.* **12**, 2166–2174 (2019).

[58] Bindhu, M. R. & Umadevi, M. Antibacterial activities of green synthesized gold nanoparticles. *Mater. Lett.* **120**, 122–125 (2014).

[59] Vanaraj, S., Jabastin, J., Sathiskumar, S. & Preethi, K. Production and characterization of bio-AuNPs to induce synergistic effect against multidrug resistant bacterial biofilm. *J. Cluster Sci.* **28**, 227–244 (2017).

[60] Nayem, S. A. et al. Green synthesis of gold and silver nanoparticles by using *Amorphophallus paeoniifolius* tuber extract and evaluation of their antibacterial activity. *Molecules* **25**, 4773 (2020). 33080946 10.3390/molecules25204773 PMC7587553

[61] Suriyakala, G. et al. Green synthesis of gold nanoparticles using *Jatropha integerrima* Jacq. flower extract and their antibacterial activity. *J. King Saud Univ. Sci.* **34**, 101830 (2022). 10.1016/j.sjbs.2021.12.007 PMC8848134 35197733

